# Occupational contact dermatitis: analysis of cases observed in a service not specialized in occupational dermatosis between 2004 and 2017^☆^

**DOI:** 10.1016/j.abd.2020.10.015

**Published:** 2021-12-04

**Authors:** Rosana Lazzarini, Mariana de Figueiredo Silva Hafner, Nathalie Mie Suzukia, Isabela Marangon Pasotti, Maria Regina de Paula Leite Kraft

**Affiliations:** a Dermatology Clinic, Faculdade de Ciências Médicas da Santa Casa de São Paulo, São Paulo, SP, Brazil; b Dermatology Clinic, Hospital Santa Casa de São Paulo, São Paulo, SP, Brazil; c Escola de Medicina da Santa Casa de São Paulo, São Paulo, SP, Brazil

Dear Editor,

Occupational dermatoses (OD) are changes in the skin, mucous membranes and adnexa, caused, maintained or aggravated by agents present at the workplace. The ODs with the greatest repercussion are occupational contact dermatitis (OCD), with an impact on the health system, workers’ remuneration, and productivity. They are the main occupational diseases in Brazil, even though they are underdiagnosed.^1^

Irritant contact dermatitis (ICD) is the most common form, restricted to areas of contact with irritants and related to the frequency and duration of this exposure. Allergic contact dermatitis (ACD), with a lower incidence, shows lesions in the areas of contact, which can spread.^1^

The diagnosis involves occupational history; synchrony between the start of the picture and exposure period; the correlation between the location of lesions and contact; improvement with absence from work and worsening with the return to it; and the patch test.^1^, ^2^, ^3^

The following were analyzed: the frequency of OCD in patients submitted to patch tests in a non-specialized outpatient clinic between 2004 and 2017; the distribution by age, sex, professional activity, and location of the dermatosis; the diagnoses; and the most common sensitizers. Data were compared with those of non-OCD patients (NOCD) (Research Ethics Committee approval, 08077219.1.0000.5479).

Data were retrospectively collected from 1,405 patients tested with the Brazilian standard test battery (FDA-Allergenic/Brazil) and fixed with appropriate retainers.

Data were analyzed using the software SPSS, version 13.0. and the results of the two groups were compared using the Chi-square test (p < 0.005).

Of the 1,405 tested patients, 349 (25.3%) were OCD and 1,031 (74.4%) were NOCD. The higher frequency of OCD when compared to a previous publication (10.9%) may reflect the increase in the number of tested substances and the involvement of Occupational Medicine in the diagnostic conclusion. However, the number is below the national frequency (34.2%) of OD cases, probably because it is a non-specialized service in these types of dermatoses.^4^, ^5^

Among the OCDs, 152 (43.6%) were ACD, 55 (15.8%) were ICD, 8 (2.3%) were atopic dermatitis (AD) and 215 (61.6%) were other dermatoses. Among the NOCD, 455 (44.1%) were ACD, 70 (6.8%) were IDC, 54 (5.2%) AD and 452 (43.8%) were other dermatoses. ACD was the most common diagnosis in both groups. However, ICD is the most common subtype worldwide, especially in occupational cases, showing a statistically higher frequency in this group (p < 0.001). The proportion of these diagnoses varies by region, due to differences in industry types, regulatory standards, notification systems, and availability of centers qualified for the performance of the patch test. On the other hand, AD can be considered an OD, since many workers experience worsening of the condition after contact with allergens and irritants in the working environment.

Among the patients with OCD, the mean age was 42.1 years, while in the NOCD it was 47.5 years (p < 0.005), resulting in losses to the production systems and social security.^1^

In the OCD group there were 182 (52.1%) women and 167 (47.9%) men, while among the NOCD there were 797 (77.3%) women and 234 (22.7%) men. The frequency of women was higher among the NOCD (p < 0.005), as they probably represent the part of the population that most often seeks medical care, but is significantly lower in the occupational group, perhaps because there are more male workers in activities subject to exposure to sensitizers and irritating substances. Moreover, women tend to be more adept at using personal protective equipment and preventive care.^6^

The most prevalent occupations in the OCD group are shown in Table 1 and involve professionals performing wet activities (contact with water >2 hours daily, or handwashing >20 times a day) or exposure to known sensitizers and irritant substances.^7^

**Table 1:** Main professional activities in patients with OCD.

Professional activity	Number	%
Housemaids/cleaning services	95	27.3
Building /bricklayer	72	20.6
Health care workers	26	7.4
Painter	12	3.4
Hairdressers	11	3.2
Others	133	38.1
Total	349	100.0

The time of disease evolution in OCD was 29.2 months, while in NOCD was 39.5 months (p < 0.005). The difficulty in maintaining working activities may have favored the earlier search for help among OCD cases.

As for the location of the dermatosis, there was a statistical difference in relation to OCD for hands (palmar and back regions) and forearms (p < 0.001), while in patients with NOCD, the face was the most affected site (582/66.7%). This difference is expected since OCD mainly affects the hands, which directly manipulate irritating or sensitizing products.

The main relevant sensitizers observed in both groups are shown in Table 2, with nickel being the most important one, present in occupational activities or not. The hands are affected due to contact with working tools such as sewing needles, tweezers, cuticle nippers, keys, and scissors.

**Table 2:** Main allergens in patients with OCD compared to those with NOCD.

Allergens	152 with OCD		455 with NOCD		
	n	%	n	%	
Nickel sulfate	98	28.1	360	35	p = 0.018
Potassium bichromate	89	25.6	87	8.4	p < 0.001
Carba mix	54	15.5	51	5.0	p < 0.001
Thiuram mix	49	14.1	41	4.0	p < 0.001
Epoxy resin	17	4.9	13	1.3	p < 0.001
Quaternium 15^a^	17	4.9	25	2.4	p = 0.021
MBT mix^b^	14	4	20	1.9	p = 0.031

a Hexamethylenetetramine chloroallyl chloride.

b Mercaptobenzothiazole.

Potassium bichromate is related to bricklayers, due to its presence in cement (as a contaminant, resulting from the manufacturing process), being an important occupational allergen in similar studies.^8^

The thiuram and carba mix and 2-mercaptobenzothiazole (MBT) groups are rubber vulcanizing agents, commonly found in gloves, used in several production sectors.

Epoxy resin is a material used in several products such as paints, floor finishes, fiberglass, adhesives for metals, wood, and concrete. Therefore, injuries occur in multiple locations and protective measures are difficult.^9^

Quaternium 15 (formaldehyde-releasing preservative), present in children's products, cosmetics, veterinary shampoos, polishing materials, and waxes, is an ubiquitous allergen.^10^

Some allergens showed high frequencies in both groups (with no statistical difference), including Kathon CG: OCD (24/6.9%) and NOCD (84/8.2%), a mixture of preservatives in aqueous products, such as cosmetics, cleaning articles (detergents, soaps, lubricating oils) and paints, with occupational cases occurring in cleaning workers, hairdressers and painters; and paraphenylenediamine, present in hair dyes: OCD (27/7.8%) and NOCD (110/10.7%), with occupational cases occurring in hairdressers, affecting the hands due to the inappropriate use of PPE.

During the study period, OCD represented 25.3% of the cases submitted to patch tests, with the group of young male individuals being the most affected, with an impact on workers' health and the country's economy. The study of OCD in the country is fundamental for the planning of preventive actions and diagnostic training in dermatological centers.

## Financial support

None declared.

## Authors’ contributions

Rosana Lazzarini: Design and planning of the study; drafting and editing of the manuscript; collection, analysis, and interpretation of data; effective participation in research orientation; intellectual participation in propaedeutic and/or therapeutic conduct of the studied cases; critical review of the literature; critical review of the manuscript; approval of the final version of the manuscript.

Mariana de Figueiredo Silva Hafner: Design and planning of the study; drafting and editing of the manuscript; collection, analysis, and interpretation of data; effective participation in research orientation; intellectual participation in the propaedeutic and/or therapeutic conduct of studied cases; critical review of the literature; critical review of the manuscript; approval of the final version of the manuscript.

Nathalie Mie Suzuki: Effective participation in research orientation; intellectual participation in the propaedeutic and/or therapeutic conduct of studied cases; critical review of the literature; critical review of the manuscript; approval of the final version of the manuscript.

Isabela Marangon Pasotti: Drafting and editing of the manuscript; obtaining, analyzing, and interpreting the data; approval of the final version of the manuscript.

Maria Regina de Paula Leite Kraft: Elaboration and writing of the manuscript; collection, analysis, and interpretation of data; approval of the final version of the manuscript.

## Conflicts of interest

None declared.
